# Endoscopic Bulking Materials for the Treatment of Vesicoureteral Reflux: A Review of Our 20 Years of Experience and Review of the Literature

**DOI:** 10.1155/2011/309626

**Published:** 2011-04-06

**Authors:** Boris Chertin, Stanislav Kocherov, Leonid Chertin, Alaeddin Natsheh, Amicur Farkas, Ofer Z. Shenfeld, Sarel Halachmi

**Affiliations:** ^1^Department of Pediatric Urology, Shaare Zedek Medical Center, Faculty of Medical Science, Hebrew University, P.O. Box 3235, Jerusalem 91031, Israel; ^2^Department of Urology, Bnai Zion Medical Center, 47 Golomb Street, Haifa 31048, Israel

## Abstract

*Purpose*. We reviewed our 20 years of experience and the current literature regarding the long-term outcome of endoscopic treatment of vesicoureteric reflux (VUR) using the different tissue bulking substances with a special emphasis on the long-term efficacy. *Material and Methods*. Our own experience and the current literature on the long-term results after endoscopic treatment using various bulking agents were reviewed. *Results*. Short-term data following endoscopic treatment of VUR is similar to the various substances and comparable in the majority of the series to the success rate following open surgery. Recently, a relatively high recurrence rate was noticed especially with the use of dextranomer hyaluronic acid (Dx/HA) as a tissue augmenting material which raises the need for further search for alternative substances. *Conclusions*. Unfortunately, there is a significant shortage of evidence-based literature on the long-term followup after endoscopic correction of reflux with various substances. No doubt, there is a high recurrence rate during long-term followup after Dx/HA injection, and there is probably lack of proper evaluation regarding the long-term efficacy of other bulking materials. These facts demand long-term close observation and long-term studies beyond the routine protocols following endoscopic treatment of VUR and the correct parental counseling upon the endoscopic correction.

## 1. Introduction

The introduction of STING two decades ago started the change in the treatment of VUR. Since the approval by the Food and Drug Administration (FDA) of Dx/HA copolymer (Deflux, Q-Med Scandinavia, Uppsala, Sweden) over the last 7 years, the endoscopic management of VUR has emerged as a first line treatment in all grades of reflux in most of the world [1–5]. The concept of the endoscopic correction of VUR offers a minimally invasive treatment in the management of UTI or renal parenchymal damage associated with reflux. The advantages of this procedure are numerous: easy to perform, short, minimally invasive, low complications rate, and short hospital stay. The overall success rates which were reported by the different groups ranged between 68–92%, depending mainly on the VUR grade [1–3, 6, 7]. The complications rate following this procedure is infrequent and relates mainly to the obstruction of ureterovesical junction (UVJ) and the development a new contralateral reflux (NCVUR) following treatment of unilateral VUR [5]. A recent meta-analysis of injection therapy considering outcomes from Dx/HA, polytetrafluoroethylene, collagen, polydimethylsiloxane, and chondrocytes suggested fewer subsequent UTIs than previously noted with open surgery or antibiotic prophylaxis, with an overall incidence of 6% (range from 2.74% to 14.15%) and febrile infection observed in only 0.75% of patients [8]. Furthermore, the recently published American Urology Association (AUA) guideline regarding VUR treatment clearly stated that febrile UTI is strongly associated with renal parenchymal damage and that successful surgical correction leads to the significant decrease of the pyelonephritis and therefore may avoid renal scarring [9]. Since endoscopic treatment of UTI has enjoyed a high rate of the success in the short-term followup, it is extremely important to address the issue of the long-term efficacy and the subsequent UTI, pyelonephritis, and renal scarring incidence following endoscopic correction of UTI. In this paper, we have summarized the worldwide experience with the long-term efficacy of the different bulking agents utilized for endoscopic correction of VUR with special emphasis on the UTI incidence and the long-term efficacy of Dx/HA copolymer as an ultimate tissue augmenting substance used for the endoscopic injection in the majority of the pediatric urology centers around the world. In addition, we have reviewed a limited experience with a new biodegradable bulking agent.

## 2. A Review of the Various Available Bulking Agents

### 2.1. Polytetrafluoroethylene (PTFE)

PTFE belongs to the group of the most widely used biomaterials in medicine. Medical applications of the PTFE include vascular grafts, heart valves, and tissue replacement patches. PTFE was one of the first widely used tissues augmenting substance for endoscopic correction of reflux [4]. Since the published experience with PTFE is over than 20 years of followup, it is interesting to observe and to compare the incidence of UTI and the long-term durability of PTFE with currently used biomaterials for injection therapy. 

The longest reported followup of children with primary VUR treated with PTFE injection was published by Chertin et al. [3]. The authors reported about 258 children with primary VUR, with a median age of 6 years comprising a total of 393 refluxing ureters. Ninety-six percent of the injected ureters had Grade III to V reflux. Eighty-eight percent of patients had a routine voiding cystourethrogram (VCUG) between 11 up to 17 years after the successful injection, showing a high success rate of 95% therefore eliminating the possibility of the silent VUR recurrence. Out of those 19 (5%) ureters, only 6 recurred showing a high-grade reflux. 

The most striking fact in this study is that none of the studied patients developed febrile UTI during and only 3.6% of children had afebrile UTI during followup. Similar findings in terms of the VUR recurrence and incidence of the febrile UTI were demonstrated by another study with a followup of 10 years after PTFE injection [4]. 

Although the majority of the pediatric urologists have acknowledged the efficacy of endoscopic correction of low-grade VUR, some still question the durability of the endoscopic correction in high-grade VUR [21]. The convincing data regarding the long-term followup over 17 years with PTFE injection in children with grade IV and V reflux was published previously [22]. In this study, 717 refluxing units were analyzed following PTFEE injection, reflux recurred only in nine units (1.2%) further showing that, with the correct choice of the implant for endoscopic correction, this procedure is durable even in high-grade VUR. 

Despite of the convincing data regarding the long-term efficacy of endoscopic correction of VUR utilizing PTFE, the concerning information regarding the possibility of the PTFE particle migration eliminated the use of PTFE for the correction of VUR. For these reasons, alternative substances have been developed (Table 1).

### 2.2. Glutaraldehyde Cross-Linked Bovine Collagen

Glutaraldehyde cross-linked bovine collagen has emerged as a first alternative to PTFE [11, 12]. Since the collagen was used very intensively in the medical industry for the manufacturing of cardiac valves and hemostatic agents, causing minimal tissue reaction when locally injected, initially it appeared as a promising substance for the VUR correction in the short term. However, long-term studies clearly showed that the initial success rate with the use of collagen dramatically decreased during long-term followup. Reunane reported his experience with collagen injection in 197 refluxing ureters in 148 children [12]. All children underwent direct radionuclide cystography at 1 month, 6 months, 2 years, and 4 years following endoscopic correction. The results for the low-grade VUR were better than previously reported about this substance demonstrating 93.9% cured rate after one month following injection; however, after 4 years only 81.8% cure rate was documented. Patients with high-grade VUR and complex cases did significantly worse with 44.4% success rates after 1 month and a very low cure rate at 4 years of only 21.4%. 

These disappointing results further demonstrated by Haferkamp et al. reporting prospectively about 36 patients treated with single-collagen injection [11]. While they have achieved 80% success at 3 months after injection, only 9% of the previously cured ureters were still free of reflux at 37 months following injections. 

Disappointing results ceased the use of Glutaraldehyde cross-linked bovine collagen for the correction of VUR.

### 2.3. Polydimethylsiloxane (Macroplastique)

Polydimethylsiloxane is a solid, elastomeric silicone, soft tissue bulking agent that has been incorporated into a patented device called Macroplastique (Uroplasty Inc., Geleen, The Netherlands) [13, 23–25]. In spite of the fact that the majority of the particles of this agent are greater than 100 *μ*m in diameter, the presence of the particles with the size of 80 *μ*m and less may still raise the fear from distant migration. The short-term results with the use of Macroplastique as a tissue augmenting substance were similar to the other bulking agents [4]. However, there is a significant shortage of prospective long-term studies, which may prove the efficacy of silicon in the treatment of VUR. Van Capelle et al. published a retrospective review on 195 patients who underwent endoscopic correction of VUR utilizing Macroplastique as a tissue augmenting substance in two institutions [24]. The study period lasted over 10 years. Overall success rate was 84% and 77%, respectively, in two departments. One of the major drawbacks of this study, as well as others dealing with long-term efficacy of the bulking agents, is that the patients were discharged from the followup after one year if they were free of symptoms. The recent review of the literature regarding long-term results of the use of Macroplastique in children with VUR has shown that after 9 years of follow up the success rate is similar to the short-term observation [26]. However, again, no precise criteria regarding the followup details such as the percentage of the patients who underwent obligatory VCUG and monitoring of the UTI were clearly stated. 

In summary, there is a lack of evidence-based data in order to give a solid report about the long-term efficacy of this substance for the correction of VUR.

### 2.4. Autologous Chondrocytes

Autologous chondrocytes were proposed by Atala and coworkers for the endoscopic treatment of VUR following successful animal experiments [14]. However, the need for two procedures under anesthesia, the first to harvest the cartilage cells for the preparation of the injection solution and the second for the endoscopic implantation, reduced the popularity of this substance. Moreover, a significant high-recurrence rate after one year raised a serious question regarding the reliability of this tissue augmenting substance in the pediatric population. For the above-mentioned reasons, this substance was abandoned.

### 2.5. Calcium Hydroxyapatite (Coaptite)

Calcium hydroxyapatite has been used as a biocompatible implant for orthopedic and dental procedures in humans for more than 25 years. In 1998, an FDA approved to study the use of Coaptite, for the urinary tract. Coaptite was used in women as a bulking agent for the treatment of female stress urinary incontinence. The initial results, safety and durability of the material for 3 years, prompted the FDA decision to approve a pilot investigation for the use of Coaptite in the treatment of VUR in children. In 2001, given initially favorable results of the pilot VUR study (70% cure in 10 patients/10 ureters at 3 months), a prospective multicenter trial of synthetic calcium hydroxyapatite, as a subureteral bulking agent, in children with traditional indications for surgical treatment of VUR was performed at 10 United States centers [15]. At 1 and 2 years, 24 out of 74 patients (32%) were cured. Ureteral cure rates were 46% and 40% at 1 and 2 years, respectively. With 35 patients treated and 85% compliance with the required 2-year VCUG, the primary center achieved 2-year cure rates of 66% of patients and 72% of ureters. 

Small study cohort and relatively short-term followup avoids the ability to give a true evidence-base-judgment about this substance as a bulking injectable agent for the treatment of VUR in children.

### 2.6. Dextranomer Hyaluronic Acid (Deflux)

Deflux is the most widely used material for the endoscopic injection of VUR, we may say that this is the most studied material and enough long-term data exist in order to understand clearly its efficacy. The substances composing Deflux are dextranomer (Dx) microspheres and nonanimal Hyaluronic Acid mixed to form viscous gel consisting of two components. Both components are made up of polysaccharides sugar-based molecules. Dextranomer microspheres are formed by cross-linking dextran polymers into porous beads of 80–250 *μ*m in diameter.

The overall success rate reported by the different groups of authors with use of Dx/HA copolymer (Deflux, Q-Med Scandinavia, Uppsala, Sweden) ranged between 68–92% depending mainly on VUR grade [1–3]. Recently, Kirsch et al. have demonstrated that by utilizing their technique of Hydrodistention Implantation Technique (HIT) injection the short-term results with the endoscopic correction may be close to those after open surgery, and in the case of low-grade reflux even similar to those following open reimplantation [1]. Therefore, the only question of Deflux long-term durability remains the subject of interest and parental concern. Unfortunately, long-term follow-up data is lacking, as well as strict criteria for what is considered a “success.” It seems that the long-term success should be defined on the basis of the prospective studies, where all patients will be required to undergo timing VCUG even after first postoperative-negative VUR imaging, strict registration of all incidences of febrile and afebrile UTIs following injection, and the record of all possible renal parenchymal changes. 

Of the available data we brought one meta-analysis that examined all types of injections, including Dx/HA, which demonstrated a primary success rate of 78.5% for grades I and II, 72% for grade III, 63% for grade IV, and 51% for grade V reflux [8]. Many of the studies included in this meta-analysis had limited followup, only single postoperative VCUG usually within the first 3 to 4 months postoperatively. The early report of 7.5 years of followup after first successful reflux correction with Deflux from a Swedish group, which pioneered the use of this tissue augmenting substance, showed very low reflux recurrence [16]. The overall success rate of 84% following first VCUG decreased to 74% in the long-term followup. Reviewing the presented data, only 45 (13.4%) out of the 334 treated ureters examined by a later VCUG performed (Table 2). Moreover, the defined of the resolution of VUR was considered as “nondilating” reflux. In those patients who had later VCUG, 96% had a continued prolonged resolution also at the 2nd and 5th years. Another study presented by Lee et al. [19] demonstrated also a relatively high incidence of VUR recurrence. The authors retrospectively studied late VUR recurrence verified by VCUG, which was performed one year after successful Deflux injection. The initial experience with Dx/HA was similar to the existing studies, with a postoperative VCUG success rate of 73%. At one year after endoscopic treatment 39 of 150 ureters exhibited VUR, resulting in a recurrence rate of 26% and an overall cumulative failure rate of 54% (130 of 241 ureters). The overall long-term success rate hence was only 46%, and only 74% of the initially successful cases remained so at within one year. Recently, the working group of pediatric urology of German Association of Pediatric Surgeons published results of the multicenter prospective trial, which aimed to evaluate the long-term efficacy of endoscopic treatment of VUR utilizing a Dx/HA [20]. A total of 284 patients (424 RRU) were treated endoscopically with Deflux injection. The reflux was corrected in 68% of all RRU. Forty-six percent of the patients completed 3 years of followup. In 21% of RRU, a relapse of VUR was diagnosed between 6 months and 3 years. Based on this data the authors strongly recommended to continue to follow of the patients even after a successful injection beyond the first 3 years after surgery. 

Renal parenchymal damage and UTI incidence following successful endoscopic correction of VUR. The treatment of VUR is lacking a based evidence protocol and remains controversial [7, 27]. Termination of the antibiotic prophylaxis in otherwise healthy child with persistent VUR at the age of 5 years or older is one of the options. Recently, Kitchens et al. published their results with this approach [28]. Out of 185 children who have been taken off antibiotic prophylaxis, 29% developed UTI. The majority was girls (91%) and the majority of UTIs were febrile. All those patients underwent surgical correction. The authors concluded that although discontinuation of antibiotics is a reasonable option, prospective randomized studies are required in order to decide whether this approach is beneficial.

## 3. Urinary Tract Infection following Injection

It has been shown that open surgical correction of VUR offers a reasonable kidney protection [29–31]. Recent data published by many researchers clearly demonstrates that subureteric Teflon injection (STING) offers a reasonable option for surgical correction of VUR. However, the question whether the surgical correction of VUR utilizing an endoscopic approach will prevent further renal damage and UTI development still needs to be answered [10, 18, 32–34]. Since we have been using the endoscopic approach over the last two decades in VUR patients, we recently published our data regarding the changes in the renal function and incidence of UTI in children who underwent successful endoscopic correction of VUR utilizing different tissue augmenting substances [10]. We have retrospectively evaluated 507 patients, 169 males and 338 females (696 renal reflux units (RRU)) with a median age of 3.7 years underwent successful endoscopic correction of primary VUR from 1988 to 2007 with a median, follow-up period of 13 years. Endoscopic correction was performed utilizing Polytetraflouroethylene (Teflon) and Dx/HA copolymer. Reflux was Grade 1 in 36 RRU (5.2%), Grade II in 178 (25.6%), Grade III in 298 (42.7%), Grade IV in 163 (23.4%), and Grade V in 21 (3.1%) renal refluxing units (RRU), respectively. DMSA scan and renal ultrasound were performed in all patients preoperatively. Renal ultrasound was performed in all patients following surgery, and Technetium 99 m dimercaptosuccinic acid (DMSA) scan was performed in 509 (73%) of the 696 RRU postoperatively. DMSA scan demonstrated renal scarring in 543 (78%) of the 696 RRU preoperatively. Renal deterioration was demonstrated in 11 of the 26 RRU with initial severe renal scarring (uptake on DMSA less than 20%). The remaining RRU from this group demonstrated insignificant change of 2.3% in the relative renal function after successful correction of VUR (*P* > .005). Those patients who demonstrated downgrading of VUR did not show new renal scars. Twenty-seven RRU (6.1%) of the remaining 446 RRU demonstrated greater than a 5% decrease in relative renal function without new scarring. Eleven children (2.2%) (8 in the Teflon and 3 in the Dx/HA copolymer group) developed febrile UTI following successful endoscopic correction, which drove a reevaluation that resulted in the diagnosis of VUR recurrence in 8 (72.7%) patients. 28 (5.6%) children suffered afebrile UTI without VUR recurrence. 

It has been shown that sterile reflux does not cause renal scars unless extreme hydrodynamic conditions exist in the sick kidney [17, 18, 34]. VUR-associated bacteriuria, which leads to a protracted inflammatory reaction, is a major cause of exudative pyelonephritis and kidney damage. In our series, only 2.2% of all the studied children developed recurrent pyelonephritis in the long-term period. However, 8 of those 11 children showed VUR recurrence on follow-up VCUG. This data is similar to the previously published one [17, 18]. Chi et al. and Sedberry-Ross et al. have evaluated the incidence of the febrile UTI following successful endoscopic correction of VUR. The Dallas group clearly demonstrated that in 159 patients, of whom 95% had preoperative urinary tract infections, and all had demonstrated a complete resolution of reflux after Dx/HA injection, 40 patients (25%) had recurrent UTI, of which half were febrile [17]. Reimaging was done in 15 patients with recurrent febrile infections, and 7 had recurrent reflux. Another study by the Washington group of 45 patients, who had undergone successful Dx/HA injection, demonstrated that 12 (27%) had recurrent urinary tract infections. On reimaging with VCUG, 10 of these patients (83%) had recurrence of VUR [18]. 

This data showed that those patients, who had shown initially VUR correction, but developed febrile UTI in the long-term followup, require prompt reevaluation in order to rule out VUR recurrence. Furthermore, in the light of recent reports regarding the low effectiveness of antibiotic prophylaxis in children with VUR, the question is raised whether we have an optimal tissue augmenting substance which has a durable long-term efficacy and a good safety profile [35–37].

## 4. New Material for Endoscopic VUR Treatment

### 4.1. Polyacrylate Polyalcohol Copolymer (Vantris)

Biodegradable elements of synthetic origin have a high rate of reabsorption after a year. Non-biodegradable agents of synthetic origin lead to the formation of a fibrotic capsule, giving stability and long-term permanence. Vantris (Promedon, Cordoba, Argentina) is categorized into this last group; it belongs to the family of Acrylics, particles of polyacrylate polyalcohol copolymer immersed in a glycerol and physiological solution carrier. Its molecular mass is very high. When injected in soft tissues, this material causes a bulkiness that remains stable through time [38].

The carrier is a 40% glycerol solution with a pH of 6. Once injected, the carrier is eliminated by the reticular system through the kidneys, without being metabolized. Particles of this polyacrylate polyalcohol with glycerol are highly deformable by compression and may be injected using a 23-gauge needle. The average of particles size is 320 mm. Once implanted, particles are covered by a fibrotic capsule of up to 70 microns. Particles of this new material are anionic with high superficial electronegativity, thus promoting a low cellular interaction and low fibrotic growth. The new polyacrylate polyalcohol copolymer with glycerol was tested for biocompatibility according to ISO 10993-1:2003 in vitro, showing that they are not mutagenic for the Salmonella T. strains analyzed. The extract turned out to be noncytotoxic for cell lines in culture and nongenotoxic for mice. in vivo studies, acrylate did not cause sensitization in mice. The macroscopic reaction of tissue irritation was not significant in subcutaneous implants and in urethras of rabbits. Seven female dogs were injected transurethrally with Vantris to evaluate short- and long-term migration (13 weeks and 12 months, resp.). No particles or signs of inflammation or necrosis are observed in any of the organs examined 13 weeks and 12 months after implantation. It seems that Vantris is meeting the criteria of the ideal tissue augmenting substance. The clinical experience with Vantris is still very limited. Eighty three patients were treated between 2005 and 2006 during multicenter trial in South America [39]. Sixty-one patients with an average age of 58 months completed a one-year followup. The number of injected ureters was 88 (27 (44%) of the patients had bilateral and 34 (55.7%) unilateral VUR). Thirty-two had grade II (36.4%), 41 grade III (46.6%), 12 grade IV (13.6%), and 3 units had grade V (3.4%), respectively. The injected volume per unit ranged from 0.2 to 1.6 mL, with a mean of 0.76 mL. The average follow-up period was 20 months with a range of 16 to 24 months. Reflux was eliminated in 78 (88.6%) kidney renal units; reflux decreased to grade I in 6 (6.8%) units and it persisted in 4 (4.6%) units, respectively. The overall success rate was of 83.6%. We have recently started in our institution a prospective trial aiming to see the short- and long-term efficacy of Vantris in children with VUR. Over the last 16 months, 59 children (22 males and 37 females), with a mean age of 9.6 years (range 1.2–18 years), underwent endoscopic treatment of VUR utilizing Vantris. VUR was unilateral in 29 (49.2%) and bilateral in 30 (50.8%) patients, comprising 59 renal refluxing units (RRU). Of those, primary VUR was in 53 (71.6%) RRU and 21 (28.4%) comprised complex cases: 5 (6.75%) duplex systems, 1 (1.4%) with Prune Belly syndrome, and 15 (20.3%) after failed previous endoscopic correction with Deflux. VUR was Grade I in 9 (12.1%), Grade II in 14 (18.9%), Grade III in 29 (39.2%), Grade IV in 17 (23%), and Grade V in 5 (6.8%) RRU, respectively. The reflux was corrected in 63 (85%) of 74 RRU, who completed 3-month followup and underwent VCUG after a first injection and after a second injection in 3 additional ureters. In 5 (6.8%) RRU reflux downgraded to grade 1; they have been taken off antibiotics and did not require any treatment. Three (4%) RRU failed first injection and are awaiting a second trial of the endoscopic treatment. Twenty one (55.3%) of the 38 patients completed a year of followup. Their US demonstrated no changes compared to the imaging performed one month after injection. In 8 (38.1%) of the 21 children who underwent one year radionuclide cystography, no reflux recurrence was shown. This short-term data shows that Vantris injection provides a high reflux resolution. However, more clinical data with longer followup is waiting with this tissue augmenting substance.

## 5. Conclusions

Many materials has been used for the endoscopic correction of VUR; although PTFE gave long-term high success rate its use was abandoned due to the fear of particle migration. All other substances did not show high success rate and are no longer in use. The currently worldwide used material for endoscopic treatment is Dx/HA. Despite short-term reported high success rate, there is a significant shortage of evidence-based literature on the long-term followup after endoscopic correction of reflux utilizing Dx/HA, the FDA approved tissue augmenting substance for endoscopic correction of VUR. However, the scanty data available clearly demonstrated that there are a high recurrence rate during the long-term followup after Dx/HA injection, which requires close observation beyond the routine protocols and correct parental counseling upon the endoscopic correction.

## Figures and Tables

**Table 1:** Long-term results of endoscopic treatment of VUR.

Author	Bulking agent	Follow-up time (years)	Short-term success rate (%)	Long-term success rate (%)
Chertin et al. [10]	PTFE	17	98.2	95
Chertin et al. [10]	PTFE	10	100	91
Haferkamp et al. [11]	Collagen	3	95	9
Reunane et al. [12]	Collagen	4	93.9	81.8
Dodat et al. [13]	Macroplastique	7	93.3	79.4
Caldamone et al. [14]	Autologous Chondrocytes	3	83	70
Mevorach et al.[15]	Coapetite	2	72	72

**Table 2:** Long-term results of endoscopic treatment of VUR with Dx/HA.

Author	Follow-up time (years)	UTI rate after injection (%)	Failure rate (%)
Läckgren et al.* [16]	7	8	13
Chertin et al.** [10]	6	2.2	3.9
Chi et al. [17]	4.8	24	12
Sedberry-Ross et al. [18]	7	27	25
Lee et al. [19]	1	None	26
Schemedding et al. [20]	3	Unknown	21

*The late VCUG was performed in only 45 out of the 334 treated ureters.

**The follow-up VCUG was not performed on a routine basis following postsurgery successful VCUG.
